# Haemoptysis as the first presentation of COVID-19: a case report

**DOI:** 10.1186/s12890-020-01312-6

**Published:** 2020-10-22

**Authors:** Elise Peys, Dieter Stevens, Yannick Vande Weygaerde, Thomas Malfait, Laurens Hermie, Philippe Rogiers, Pieter Depuydt, Eva Van Braeckel

**Affiliations:** 1 Department of Internal Medicine and Paediatrics, Ghent University, Ghent, Belgium; 2 Department of Respiratory Medicine, Ghent University Hospital, Corneel Heymanslaan 10, 9000 Ghent, Belgium; 3 Department of Radiology, Ghent University Hospital, Ghent, Belgium; 4 Department of Respiratory Medicine, Sint-Lucas Hospital, Bruges, Belgium; 5 Department of Intensive Care Medicine, Ghent University Hospital, Ghent, Belgium

**Keywords:** COVID-19, Haemoptysis, Alveolar bleeding, Bronchoalveolar lavage, Case report

## Abstract

**Background:**

Coronavirus disease 2019 (COVID-19), caused by the severe acute respiratory syndrome coronavirus 2 (SARS-CoV-2), is an ongoing pandemic that profoundly challenges healthcare systems all over the world. Fever, cough and fatigue are the most commonly reported clinical symptoms.

**Case presentation:**

A 58-year-old man presented at the emergency department with acute onset haemoptysis. On the fifth day after admission, he developed massive haemoptysis. Computed tomography (CT) angiography of the chest revealed alveolar haemorrhage, more prominent in the left lung. Flexible bronchoscopy confirmed bleeding from the left upper lobe, confirmed by a bronchial arteriography, which was successfully embolized. Nasopharyngeal swabs (NPS) tested for SARS-CoV-2 using real-time polymerase chain reaction (RT-PCR) repeatedly returned negative. Surprisingly, SARS-CoV-2 was eventually detected in bronchoalveolar lavage (BAL) fluid.

**Conclusions:**

Life-threatening haemoptysis is an unusual presentation of COVID-19, reflecting alveolar bleeding as a rare but possible complication. This case emphasises the added value of bronchoscopy with BAL in the diagnostic work-up in case of high clinical suspicion and negative serial NPS in patients presenting with severe symptoms.

## Background

Coronavirus disease 2019 (COVID-19) caused by the severe acute respiratory syndrome coronavirus 2 (SARS-CoV-2) is an ongoing pandemic that profoundly challenges healthcare systems all over the world. Scientific evidence on this new virus is growing and the clinical characteristics are increasingly understood. Among COVID-19 patients, fever, cough and fatigue are the most commonly reported clinical symptoms. The main reason for hospitalisation is the onset of acute hypoxemic respiratory failure [1].

Haemoptysis is rarely reported as a symptom of COVID-19. Only very few cases have been described in literature [1, 2]. Here, we report an unusual case of a man who presented with life-threatening haemoptysis as the first and unique symptom of COVID-19.

## Case presentation

In the spring of 2020, a 58-year-old man with moderate chronic obstructive pulmonary disease (COPD) presented at the emergency department of the referring regional hospital with moderate haemoptysis of acute onset. He reported no fever, chills, chest pain or worsening dyspnoea. A recent extensive diagnostic work-up for unintentional weight loss was negative for cancer. The patient was a 30 pack-year current smoker. He frequently used nicotine-containing electronic cigarettes (e-cigarettes) and had smoked marijuana occasionally until a few months before admission. He reported no other relevant medical history. He worked as a librarian and lived with his wife in a rural area. Three weeks before admission he had cleaned a dried-up fishpond. His medication included inhaled formoterol, which he only used as needed, and occasionally ibuprofen.

At admission the patient’s temperature was 36.2 °C, blood pressure 163/79 mmHg, heart rate 95 beats per minute and oxygen saturation on pulse oximetry 87% at ambient air. He appeared comfortable, with no signs of respiratory distress. The lungs were clear on auscultation and the heart rhythm was regular without murmurs.

Laboratory analysis showed a normal white blood cell count and C-reactive protein (CRP) and a haemoglobin level of 13.2 g/dL (reference range, 13.2 to 16.8). D-dimers were not elevated. Renal function and bilirubin were normal, as were the levels of the liver enzymes. Anti-neutrophil cytoplasmic antibodies (ANCAs) directed against proteinase 3 (PR3-ANCA) and myeloperoxidase (MPO-ANCA) were negative, as well as antibodies against glomerular basement membrane, antinuclear and antiphospholipid antibodies. Arterial blood gas analysis revealed moderate hypoxemia with a partial pressure of oxygen (P_a_O2) of 60 mmHg (reference range, 83–108 mmHg) and a partial pressure of carbon dioxide (P_a_CO2) of 37 mmHg (reference range, 35–45 mmHg) resulting in an elevated alveolar – arterial gradient of 43.5 mmHg (reference estimated age-specific gradient, 17 mmHg).

**Fig. 1:**
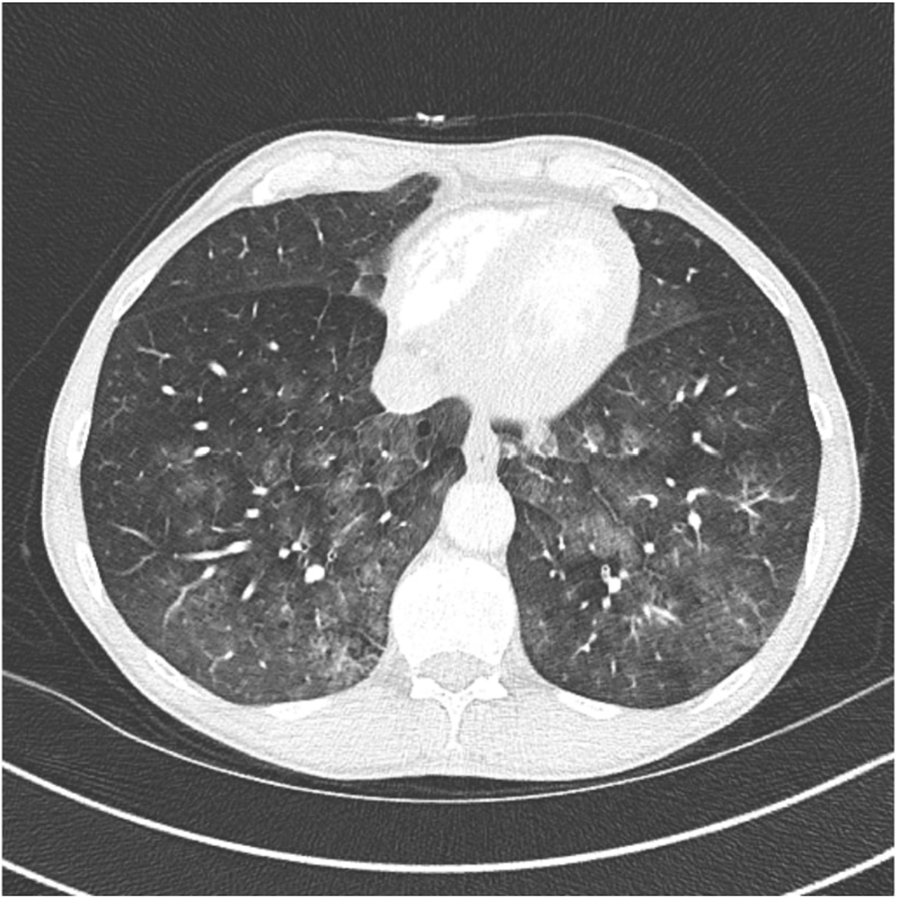
CT showing central ground glass opacities in the lower lobes, suggesting DAH

On computed tomography (CT) angiography of the chest, centrally distributed ground glass opacities were seen in the lower lobes, suggesting diffuse alveolar haemorrhage (DAH), as well as centrilobular emphysema in the upper lobes (Fig. 1). There was no evidence of pulmonary embolism.

According to the institutional guidelines during the current COVID-19 pandemic, nasopharyngeal swab (NPS) samples on two consecutive days were obtained and tested for SARS-CoV-2 using real-time polymerase chain reaction (RT-PCR), which repeatedly returned negative.

Flexible bronchoscopy performed on the third day revealed numerous blood-tinged secretions without signs of active bleeding. After clearing of the secretions, the bronchial tree appeared normal without structural abnormalities. BAL specimens were obtained for culture and pathology. To explore the possibility of e-cigarette or vaping product use-associated lung injury (EVALI) and recent use of marijuana, vitamin E acetate was tested in the BAL fluid, but was reported negative.

Surprisingly, SARS-CoV-2 was detected in the BAL fluid by means of RT-PCR. Based on the current insights at that time and in accordance with the national guidelines, the patient was started on hydroxychloroquine (400 mg twice daily at day 1, followed by 200 mg twice daily at day 2 to 5) along with amoxicillin/clavulanic acid and azithromycin (QTc 429 ms before treatment initiation). Remdesivir, tocilizumab and convalescent plasma were not available at that time, except through clinical trials and urgent medical need programs for which the patient did not qualify.

On day four, the patient was subsequently transferred to the COVID-19 respiratory intermediate care unit of our hospital. Upon admission in our centre, the patient was in mild respiratory distress. His temperature was 38.0 °C, blood pressure 107/67 mmHg, heart rate 106 bpm and oxygen saturation on pulse-oximetry 92%, while receiving supplemental oxygen through a nasal cannula at a flow rate of two litres per minute. The lungs were clear on auscultation. Further physical examination was normal. The white blood cell count was 6070/μL (reference range, 3650–9700/μL), with a lymphocyte count of 730/μL (reference range, 1133–3105/μL). The platelet count was 137,000/μL (reference range, 149,000-319,000/μL), the hemoglobin level 10.1 g/dL (reference range, 12.9–17.3 g/dL), the ferritin level 462 μg/L (peak value: 608 μg/L at day 10) (reference range, 20–280 μg/L), the total bilirubin level 2.1 g/dL (reference range, 0.2–1.3 g/dL), the aspartate aminotransferase level 335 U/L (reference range, 0–37 U/L), the alanine aminotransferase level 360 U/L (reference range, 7–40 U/L) and the C-reactive protein 12.1 g/L (reference range, < 5 g/L). Renal function was normal, as was the coagulation screen with a prothrombin (PT) level of 79% (reference range, 70 to 120), an activated partial thromboplastin time (aPTT) of 36 s (reference range, 28.9 to 38.1) and D-dimers of 880 ng/mL (reference range < 270 ng/mL). Tests for *Legionella pneumophila* serogroup 1 and *Streptococcus pneumoniae* antigens in the urine were negative, as well as leptospirosis serology. Pulmonary hypertension was ruled out by transthoracic echocardiography.

On the fifth day of hospitalisation, the patient developed sudden massive haemoptysis and the oxygen saturation dropped to 82% despite the patient was receiving 15 l per minute supplemental oxygen through a non-rebreathing mask with reservoir bag. An urgent intubation was performed and continuous infusions of propofol, remifentanil and norepinephrine were initiated. A new CT angiography of the chest was done, revealing an alveolar haemorrhage, more prominent in the left lung. Flexible bronchoscopy performed at the intensive care unit revealed a blood clot obstructing the left main bronchus. After manipulation and retraction of the clot, bleeding recommenced and appeared to initiate from the left upper lobe. Local iced saline and epinephrine were administered. Because of persistent bleeding, a bronchial arteriography was performed, showing an inflammatory blush arising from the left bronchial artery and from the left branch of the right bronchial artery, which were successfully embolized.

**Fig. 2:**
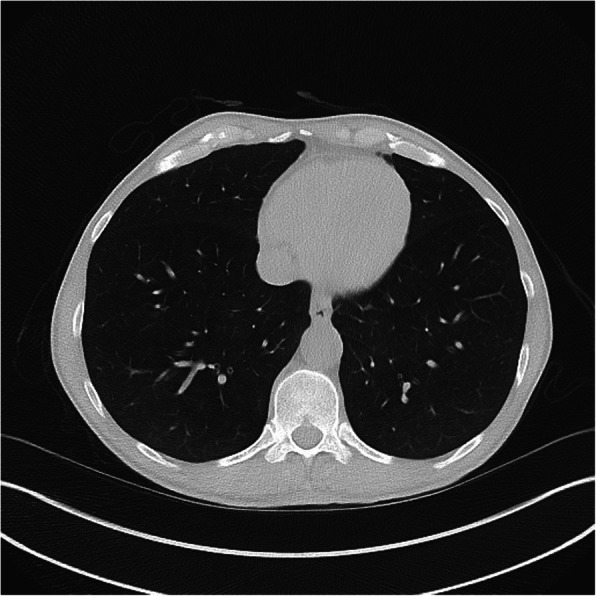
Follow up chest CT showing complete resolution of the ground glass opacities

The patient was extubated the next day and recovered fully. Follow up chest CT two weeks later showed complete resolution of the ground glass opacities (Fig. 2). Given the fact that no other aetiology was found, COVID-19 was considered to be the main cause of this patient’s haemoptysis.

## Discussion and conclusions

The diagnosis of COVID-19 can sometimes be challenging due to a nonspecific clinical presentation. Cough, fever and fatigue are the most commonly reported clinical symptoms. Unusually, in this case, haemoptysis was the initial and unique symptom of SARS-CoV-2 infection in a patient with underlying emphysema. Haemoptysis is the expectoration of blood or blood-tinged sputum from the respiratory tract. It is considered life-threatening when it causes clinical consequences such as respiratory failure from airway obstruction, as in this case, or hypotension [3, 4]. In Europe, bronchiectasis, malignancies, post-tuberculosis sequelae and idiopathic bleedings have been recognised as the most frequent causes over the last decade [5]. Other, more rare aetiologies, are pulmonary embolism, vasculitis, coagulation disorders, and arteriovenous malformations [4]. None of these were present in this patient. Since the patient frequently used e-cigarettes, EVALI was briefly considered but discarded when the diagnosis of COVID-19 was established [6].

To date, haemoptysis has rarely been described as a symptom of COVID-19. In a large case series including 1099 hospitalised patients with laboratory confirmed COVID-19 in China, haemoptysis occurred in ten patients (0.9%) [1]. Fu and colleagues on the other hand performed a systematic review and meta-analysis of the clinical characteristics of COVID-19 involving 43 studies and showed a prevalence of 2% [2]. However, the severity of haemoptysis was not mentioned. In two other case reports, haemoptysis was described as a symptom of a SARS-CoV-2 infection. In the case presentation of Shi et al. [7], haemoptysis was the only clinical symptom during the first ten days of the disease course, whereas Casey and co-workers presented a case of COVID-19 associated with acute segmental pulmonary emboli which eventually caused haemoptysis [8]. The latter demonstrates the highly thromboembolic risk related with this disease [9]. It is not known whether alveolar capillary microthrombi contributed to the pulmonary haemorrhage in this patient.

To our knowledge, this is the first case of COVID-19 associated with life-threatening haemoptysis, initially thought to be a diffuse alveolar haemorrhage (DAH). Follow-up chest CT after two weeks showed complete resolution of the ground glass opacities, reinforcing the hypothesis that these opacities were caused by alveolar bleeding rather than COVID-19 pneumonia. Other infectious diseases have been linked with alveolar haemorrhage in immunocompetent patients including influenza A (H1N1), dengue, malaria, *Staphylococcus aureus* infection and leptospirosis [10]. The latter was explicitly ruled out in this case because of the exposure history and the concomitant thrombocytopenia and elevated hepatic transaminases.

In this case, the diagnosis of COVID-19 was established by means of SARS-CoV-2 RT-PCR on BAL fluid whereas two consecutive NPS were negative. A recent study showed that BAL fluid yielded the highest positive rates suggesting a higher sensitivity than NPS [11]. To minimize the risk of transmission of infection to health care workers, bronchoscopy only has a limited role in the diagnosis of COVID-19 [12]. However, it can have an added value in establishing alternative microbiological diagnoses [13]. Furthermore, in the current ongoing pandemic setting, including SARS-CoV-2 PCR in every BAL specimen obtained for diagnostic workup of unexplained pulmonary pathology might be warranted.

In summary, life-threating haemoptysis can be the first presentation of SARS-CoV-2 infection. This case illustrates alveolar bleeding as a possible complication of COVID-19. In this patient, bronchoscopy with BAL had an added diagnostic value. In patients with severe symptoms and of high clinical suspicion despite negative NPS, bronchoscopy with BAL should be considered in the diagnostic work-up. Despite all current knowledge of COVID-19, it is still a novel disease and clinicians should be careful when patients present with respiratory symptoms of unknown aetiology.

## Data Availability

Data sharing is not applicable to this article as no datasets were generated or analyzed during the current study.

## Abbreviations

ANCA: Anti-neutrophil cytoplasmic antibodies
aPTT: Activated partial thromboplastin time
BAL: Bronchoalveolar lavage
COPD: Chronic obstructive pulmonary disease
COVID-19: Coronavirus disease 2019
CRP: C-reactive protein
CT: Computed tomography
DAH: Diffuse alveolar haemorrhage
e-cigarettes: Electronic cigarettes
EVALI: E-cigarette, or vaping, product use-associated lung injury
MPO-ANCA: Anti-neutrophil cytoplasmic antibodies directed against myeloperoxidase
NPS: Nasopharyngeal swab
PaCO2: Partial pressure of carbon dioxide
PaO2: Partial pressure of oxygen
PR3-ANCA: Anti-neutrophil cytoplasmic antibodies directed against proteinase 3
PT: Prothrombin time
RT-PCR: Real-time polymerase chain reaction
SARS-CoV-2: Severe acute respiratory syndrome coronavirus 2
